# Iterative immunoprecipitation and phage pre-wash dramatically improve epitope-resolved serology by VirScan

**DOI:** 10.3389/fviro.2026.1761741

**Published:** 2026-03-24

**Authors:** Lily Kjendal, Chase Whelihan, Olivia Garvin, Benjamin Will, Ian Lee, Zachary D. Miller, William Dowell, Jacob Dearborn, Sylvester Languon, Tylar Kirch, Zachary R. Miller, Sophie Roy, Olivia Evans, Devdoot Majumdar

**Affiliations:** 1 Department of Surgery, Larner College of Medicine, University of Vermont, Burlington, VT, United States,; 2 Cellular, Molecular, and Biomedical Sciences Program, Burlington, VT, United States

**Keywords:** antibody repertoire, epitope mapping, iterative immunoprecipitation, phage pre-wash, PhIP-seq, systems virology, viral serology, VirScan

## Abstract

Accurate mapping of antibody epitope repertoires is essential for understanding infection, vaccination, and immune history. Phage immunoprecipitation sequencing (PhIP-seq), including the widely used VirScan platform, offers single - peptide resolution across the human virome; however, such measurements are sometimes beset with limitations stemming from weak signal-to-noise ratio, non-specific phage binding, and inconsistent peptide enrichment. With such methods, it is imperative to differentiate true antibody-antigen interactions from background noise. Here, we systematically evaluate the impact of key experimental variables on assay performance and identify two synergistic modifications that markedly improve epitope-level viral serology: (1) iterative rounds of immunoprecipitation and (2) serum pre-washing with wild-type phage. Across healthy donor sera, pooled HIV-seropositive sera, and influenza-vaccinated rabbit sera, the optimized workflow produces a substantial expansion of the enriched peptide population, significantly higher normalized peptide counts, and improved separation of viral epitopes from background noise while preserving global library representation. The protocol enables quantitative detection of HIV epitopes across a 100-fold dilution series, resolving immunodominant gp160 regions, and identifies strain-specific hemagglutinin epitopes elicited by influenza vaccination, including expected public stem-directed linear epitopes. These results provide a reproducible, generalizable workflow that enhances viral epitope discovery, serosurveillance sensitivity, and vaccine-response mapping. This optimized PhIP-seq framework strengthens the utility of VirScan in systems virology by enabling more accurate and quantitative inference of viral antibody repertoires.

## Introduction

Phage immunoprecipitation sequencing, known as PhIP-Seq, is a powerful tool that allows for high throughput assessment of antibody repertoires (1, 2). By screening libraries of bacteriophage, cloned with pooled oligonucleotide technology to display hundreds of thousands of antigens against small volumes of serum, the valence and targets of many clinical antibody responses have been profiled (3–11). Focusing on the human virome, the VirScan library displays over 115, 000 epitopes in 56-amino acid tiled windows and can be used to detect global, virus-specific, and protein-specific humoral immune responses (4, 12–16). By combining phage display with next generation sequencing (NGS), this antibody–antigen detection can be scaled to large libraries and population level serum cohorts (17, 18).

The high throughput nature of these approaches has yielded findings about the antigenicity of multiple viruses and bacteria in large clinical cohorts. However, despite its scalability, this method cannot simply be equated to performing hundreds of thousands of traditional ELISAs at once. Several intrinsic differences exist with PhIP-seq which affect its performance and relevant applications, including: a lack of conformational epitopes and post-translational modifications in displayable epitopes, an introduction of competition with other antigen–antibody pairs, and statistical shot noise making it challenging to reliably identify weak antibody signals amidst background noise (2). Acknowledging these certain advantages to traditional ELISAs, it must also be noted that the milieu of antibodies competing to bind a large diversity of antigens in concert is one attribute where VirScan mimics the *in vivo* complexity of antibody–antigen binding. In this situation, availability of antibody to bind to antigen (presented on phage), akin to a physiological situation.

Over the years, multiple variations of PhIP-seq have emerged to address some of these challenges. Some approaches improve signal detection by using multiple successive rounds of IP, similar to traditional phage display panning which typically involves 2–5 rounds to enrich binders (2, 19). Others focus on enhancing specificity by optimizing the ratio of antibody to library, among various other protocol tweaks (6). While all this works towards better sensitivity and specificity, it has also been a priority to improve the scalability of the process by implementing methods to make the labor-intensive process effective on a larger scale (14).

In addition, numerous analytical approaches have been developed to parse PhIP-seq data into clear serological readouts using machine learning, RNA-seq adapted software (AVARDA), Gaussian distribution modeling, and z-score-based viral scores (20–23). Furthermore, pipelines have been developed to optimize the inherent redundancies of the method, limiting the cloned epitopes to those that most efficiently and accurately reflect seropositivity (24).

In practice, routine PhIP-seq-based assessment of virome-specific antibodies entails data curation, secondary library creation, and often employs external focuses to identify rare outliers. Assessment of peptide-level reactivities is surprisingly uncommon, despite the attractive simplicity of basic enrichment metrics. Here, we refine and validate the popular VirScan PhIP-seq approach with a series of protocol optimizations. Specifically, utilization of pre-wash and iterative rounds of immunoprecipitation and amplification are found to improve the signal-to-noise ratio and allow for highly specific characterization of epitopes. Together with the analytical approaches described above and careful assessment of limitations, we propose a general approach to an optimized PhIP-seq protocol using the VirScan library.

## Results

In order to determine whether “enriched peptides” would be immediately evident from a single sample of healthy human control serum (denoted Human Control 1), we first performed an initial implementation of VirScan PhIP-seq protocol. Briefly, this protocol implemented an incubation of the VirScan phage library with serum before magnetic pulldown of antibody-bound phage in a single round of immunoprecipitation. Antibody-bound peptide epitopes were then identified via PCR-amplification of the displayed peptide insert and NGS. To control for unevenness in phage library representation, sequencing of 1μL unprecipitated, stock library phage validated library quality, yielding 98.44% representation with relatively even distribution and minimal dominant outliers (Supplementary Figure 1).

**FIGURE 1:**
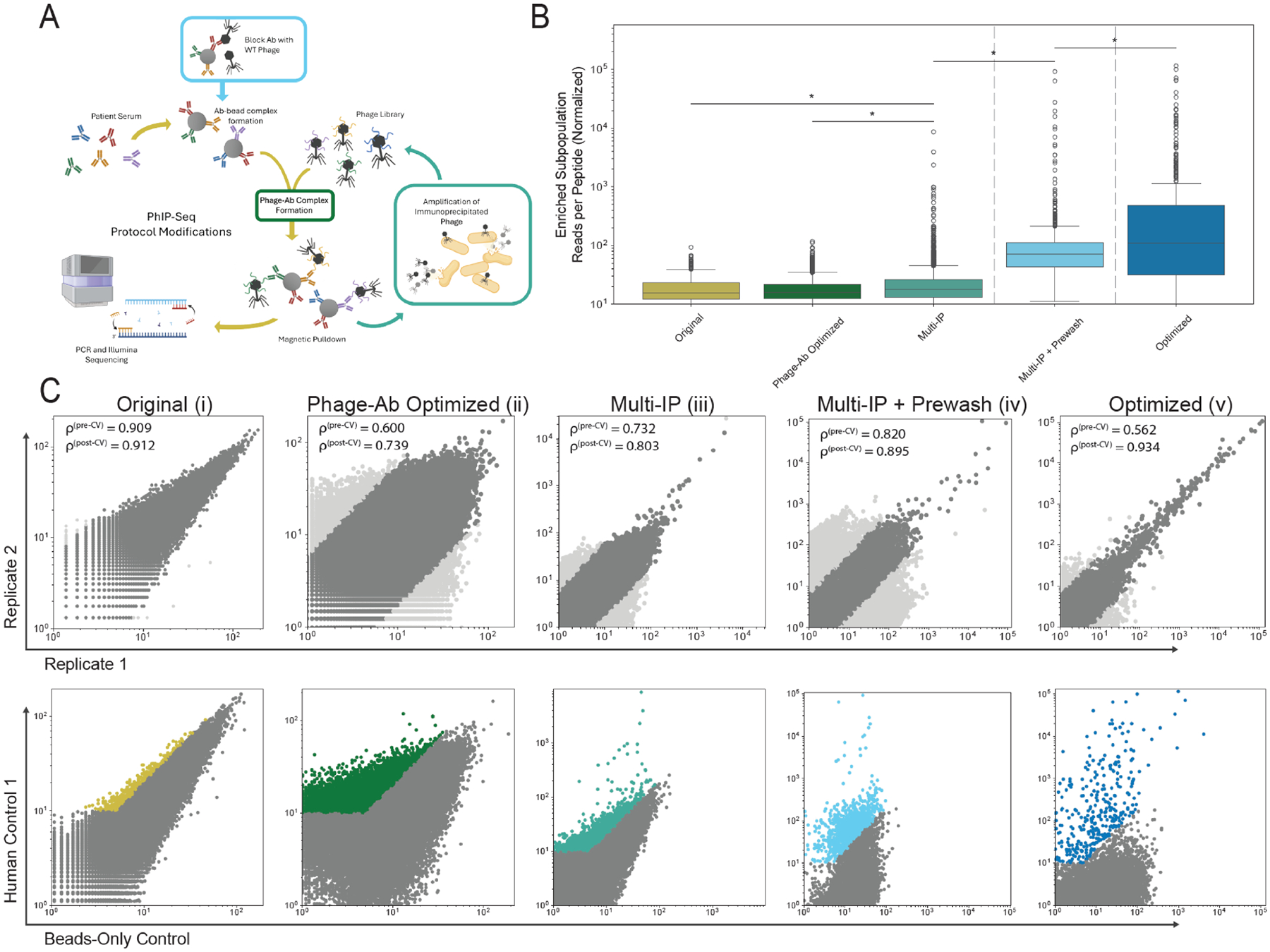
Multiple rounds of immunoprecipitation and phage pre-wash enhance peptide enrichment. (**A**) PhIP-seq method modifications tested in each iteration (yellow: original protocol, green: phage-Ab optimization, teal: multiple rounds of immunoprecipitation (Multi-IP), cyan: multiple rounds of immunoprecipitation and phage pre-wash (Multi-IP + Pre-wash)). (**B**) Distributions of enriched peptide counts for each tested protocol (post-normalization per million reads, enrichment conditions include CV < 100, normalized count > 10, and fold-change > 2 over mock-IP). (**C**) Peptide outliers are excluded in CV thresholding of serum replicates (top row). Remaining peptide counts are plotted in comparison to mock-IP peptide counts (normalized to counts/million reads), enriched peptides are shown in color (bottom row). * Means: P value < 0.05.

Surprisingly, using this protocol on healthy control serum, no peptides from the VirScan library were visibly enriched (Figure 1C(i)). Data quality was validated by assessment of reproducibility (via Spearman’s rank correlation coefficient), and enrichment was defined by a peptide having at least an average of 10 normalized counts and a fold change over bead-only control of 2 as well as being within the coefficient of variation threshold of 100. This metric for enrichment (Figure 1C(i)) did not yield a distinct population when overlayed on the entire dataset, despite the expectation that vaccination and other common epitopes from natural infection would yield a distinct virus-specific subpopulation in healthy control serum.

To facilitate improved detection of an enriched population, we implemented a series of optimizations of the PhIP-seq protocol (Figure 1A): titration of antibody:phage ratio (Figure 1C(ii)), iterative immunoprecipitation (Figure 1C(iii)), and the blocking of beads with wild-type phage (Figure 1C(iv)). These modifications were undertaken in pursuit of an optimized protocol resulting in a clearly defined enriched population (marking virus-specific epitopes) as compared to beads-only “mock-IP” control.

First, we modified the phage:antibody ratio, increasing the quantity of antibody added and decreasing the quantity of library, as shown to be efficacious in other manifestations of PhIP-Seq (6). Notably, the reproducibility between samples decreased from the original protocol as measured by Spearman’s rank correlation coefficient, r: pre-CV thresholding going from 0.909 to 0.600 and post-CV thresholding going from 0.912 to 0.739 (Figure 1C(i–ii)). For transparency, the number and percentages of peptides excluded via CV thresholding is outlined in Supplementary Table 1. There was no significant difference found between the enriched populations of the initial protocol and the Phage-Ab optimized protocol (Figure 1B).

In order to permit better separation of enriched peptides, a panning-like approach can be taken, wherein the eluted phage from one round of immunoprecipitation (with presumably higher abundance of bound displayed antigens) is amplified and used as the input for the subsequent round. This process can then be repeated iteratively, honing the library distribution of the subsequent inputs towards phage displaying only target antigens (25).

Upon testing the effects of multiple rounds of immunoprecipitation (Figure 1C(iii)), a small population of peptides created a distinct subpopulation as compared to the larger pool of peptides, suggesting increased detection of viral target peptides. While this increase was found to be significant when comparing normalized counts of the enriched peptides to the original and phage-antibody optimized protocols (Figure 1B), the enriched subpopulation was nevertheless sparse.

A wildtype phage pre-wash was proposed to reduce the effects of antibody-to-phage non-specific binding (26). By allowing serum to incubate with wildtype phage before being exposed to library-displaying phage, any phage-specific or non-specific binding could occur without resulting in the amplification of non-target epitope inserts. Indeed, the previously sparce distinct subpopulation of enriched peptides became markedly more populated (Figure 1C(iv)) and had significantly increased normalized counts when compared to all previous protocol iterations (Figure 1B).

Finally, with all modifications combined, the volume of beads used was scaled 8-fold (from 5uL to 40uL) to increase the overall phage capture of the pulldown (Figure 1C(v)). When combined, these optimizations significantly increased normalized counts of enriched peptides when compared to all previous iterations (Figure 1B). Taken together, these optimizations collectively improved detection and enrichment of antibody-bound peptides. To ensure the increase in enrichment corresponds with reasonable viral hits, pathogen-specific interactions were assessed across optimizations, with top pathogen hits of the optimized protocol including Rhinovirus A, *Streptococcus pneumoniae*, and Human herpesvirus 4/5 (Supplementary Figures 3A, B).

To further test the improvements of the optimized protocol, the use of a serum of known HIV seropositivity was employed. In our preliminary work with the optimized protocol, we detected enriched peptides in healthy human control serum; peptides from the enriched population can then be sorted by organism and protein to further characterize the epitope binding of antibodies to the VirScan library. To test the limits of our optimized protocol, we used samples from a public biorepository (BEI) of pooled HIV (Polyclonal Anti-Human Immunodeficiency Virus Immune Globulin, Pooled Inactivated Human Sera at 50mg/ml ig) serum from elite controllers of HIV infection. This concentrated, verified HIV seropositive serum was serially diluted into commercially available human control serum (denoted Human Control 2) with the goal of validating the specificity and sensitivity of our optimized protocol.

**FIGURE 2:**
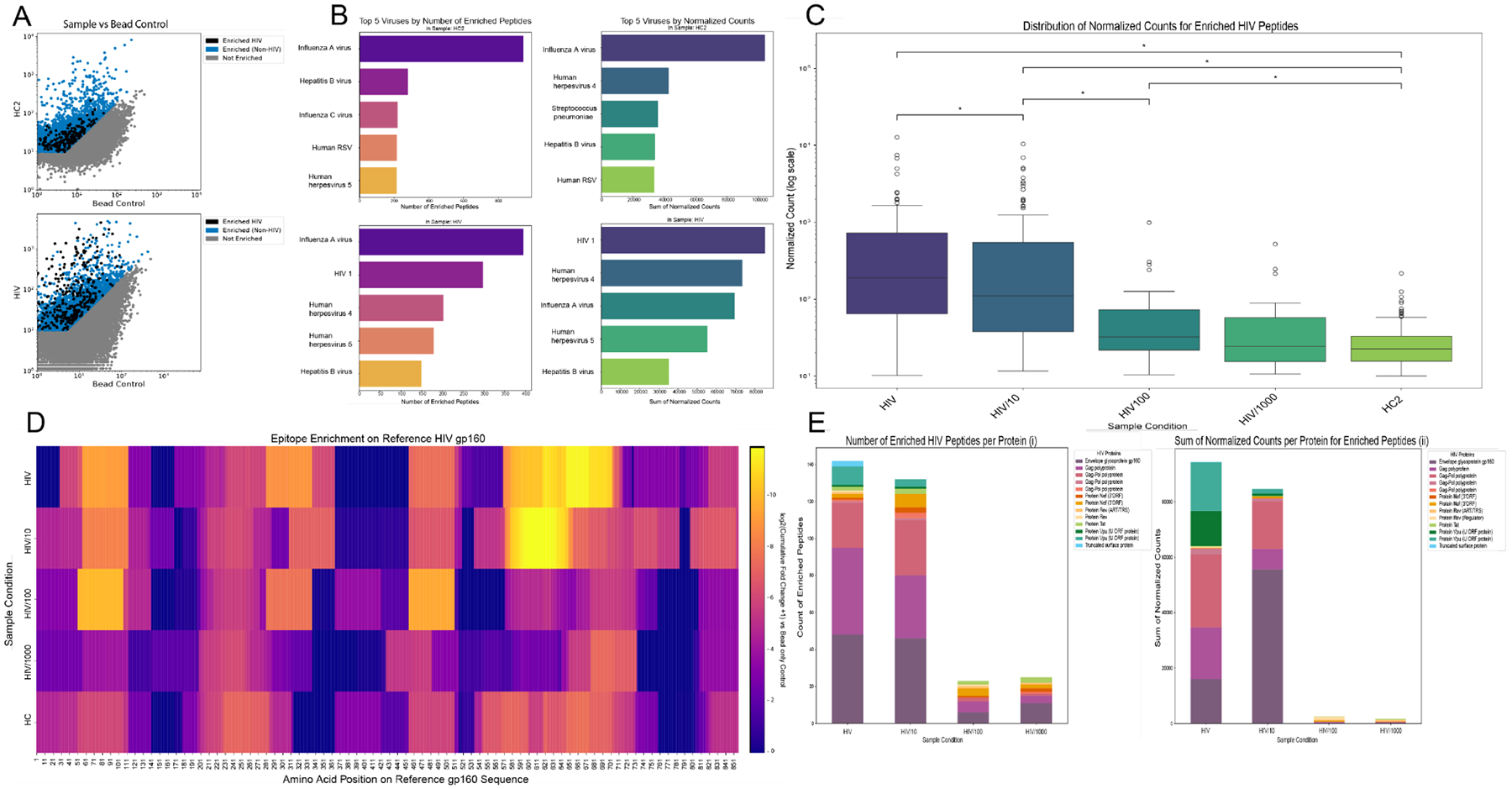
The optimized protocol can detect varied enrichment level of diluted pooled HIV seropositive serum. (**A**) Normalized counts of peptides with a sufficient CV are plotted against the bead-only control for a healthy human control, and a pooled HIV seropositive serum. Enriched peptides are shown in light blue, peptides below the threshold of enrichment are shown in grey, and black dots represent HIV-1peptides from multiple strains of the virus. (**B**) The top 5 pathogens by total normalized counts and the number of enriched peptides for Both healthy human and HIV sera. (**C**) Distribution of normalized counts for only enriched HIV peptides for each HIV sera dilution (HIV 1:10, HIV 1:100, HIV 1:1000, diluted in healthy human control serum: HC2) and healthy human control HC2. (**D**) Log2 fold change of normalized counts vs input for the aligning HIV peptides along the 851 amino acid sequence of HIV’s GP160 protein, for each HIV sera dilution and HC2. Log2 foldchange of normalized counts vs. healthy human control for all HIV dilutions. (**E**) Protein-level breakdown of HIV-1 enrichment for each dilution of HIV in total number of enriched peptides(i), and when accounting for normalized count (ii). * Means: P value < 0.05.

Both the human control and HIV serum samples display enrichment of HIV-associated peptides based on our thresholds (Figure 2A). However, there is a significant increase in the normalized counts of the enriched HIV peptides in the HIV sample when compared to Human Control 2 (Figure 2D).

When comparing ranked lists of virus hits by total normalized count of their enriched peptides (Figure 2B(i)), highly serologically represented viruses such as Influenza A Virus (4) were present in both the Human Control 2 and HIV serum samples. HIV was the clear top-ranking virus by this metric within the HIV sample, while being absent in Human Control 2. When the list is ranked based on the number of enriched peptides, without factoring in their individual counts (Figure 2B(ii)), HIV had the second highest score within the HIV sample, behind only Influenza A Virus, while once again not being present in the human control plot, as expected. This shows that not only is HIV seropositivity detected, but the signal strength of such a concentrated sera pool of elite protectors is reflected in its place amongst other potential viral targets (27).

To assess the lower limit of antibody detection using our optimized protocol and analytical thresholds, HIV serum was serially diluted (undiluted, 1:10, 1:100, and 1:1000) in Human Control 2 serum. As expected, HIV-specific enrichment decreased with increasing dilution (Figure 2C). Notably, statistical testing revealed significantly increased HIV enrichment when compared to Human Control 2 serum in all dilutions, except for 1:1000.

We used an epitope mapping approach to characterize the antibody binding landscape within a single protein, in this case HIV gp160, the precursor to HIV’s envelope proteins. Here, the primary question was whether concentration of HIV serum would impact epitope specificity. Plotted as a colormap of the enrichment (log2 fold change over human control of HIV peptides aligned to HIV gp-160 sequence), enriched regions were revealed along the gp-160 peptide for all samples, though epitopes in residues 570–700 visibly decrease after the 1:10 dilution, and epitopes in residues 50–110 and 310–330 visibly decrease after the 1:100 dilution (Figure 2D) (28).

**FIGURE 3:**
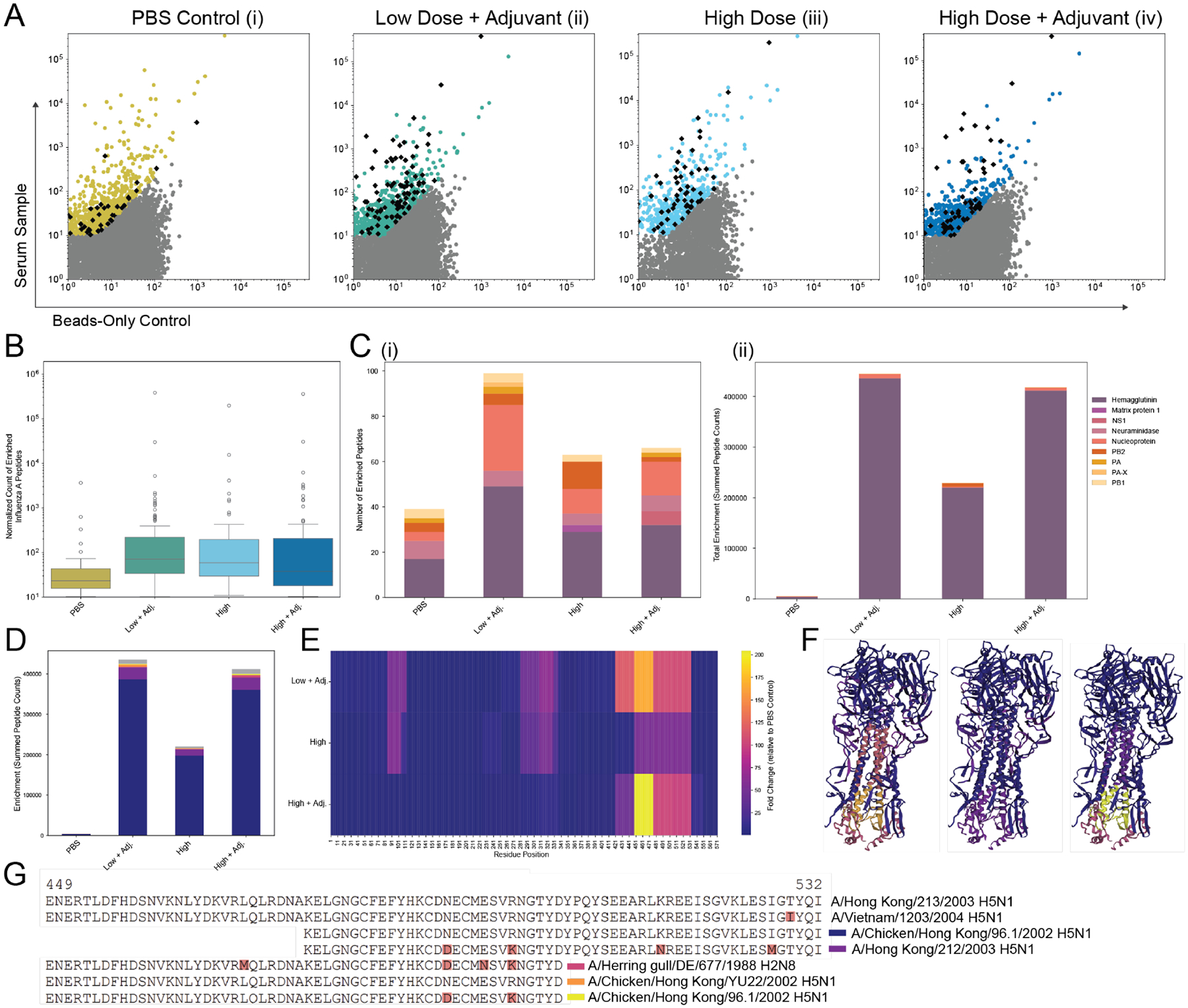
Optimized protocol distinguishes vaccinated from unvaccinated sera and detects vaccine-induced epitope specificity. (**A**) Normalized peptide counts with sufficient CV are plotted against beads-only control for four rabbit serum samples post immunization, immunized with PBS control, low dose Influenza A virus vaccine with adjuvant, high dose vaccine, and high dose vaccine with adjuvant (enriched peptides shown in color: yellow, teal, light blue, and dark blue, respectively, and enriched Influenza A peptides shown as black diamonds). (**B**) Distributions of enriched Influenza A peptide counts for sera. (**C**) Protein-level breakdown of Influenza A enrichment for each sample in total number of enriched peptides (i), and when accounting for normalized count (ii). (**D**) Peptide-level breakdown of hemagglutinin enrichment with top five peptides shown in blue, purple, pink, orange, and yellow, respectively, and identified in the adjacent table. (**E**) Heatmap of residue-level relative hemagglutinin enrichment (normalized to PBS control) of each vaccinated sample. (**F**) Relative enrichment of each vaccinated sample mapped onto 3D model of H5N1 Influenza A virus hemagglutinin protein. (**G**) Alignment of vaccine strains and top peptides (mismatches highlighted in red).

Further analysis of enriched peptides between HIV dilutions shows diverse representation of HIV proteins. We employed 2 metrics to analyze per protein peptide diversity for that virus; summed normalized counts and number of enriched peptides (seen in Figures 2E, 3C). The aim of these metrics is to paint a clearer picture of where these peptides are actually binding throughout the viral proteome. Sum of enriched counts gives us a figure on the number of binding events per protein, while the number of enriched peptides gives us information on the diversity of binding events. The plotting of proteins by number of enriched peptides shows the representation of 13 different HIV proteins, top hits including: envelope glycoprotein gp160, gag polyprotein, and gag-pol polyprotein (Figure 2E(i)). When plotting by another metric, total normalized count, the same 3 proteins were represented heavily with the notable additional inclusion of protein VPU for the undiluted sample (Figure 2E(ii)). The undiluted and 1:10 dilution showed relatively similar peptide profiles in both metrics, but when further diluted the protein representation dropped off.

We then asked, using a common viral target—Influenza A—if vaccine-elicited antibody targets could be detected quantitatively, and if those induced antibody repertoires were distinct across various dosing strategies. We used our optimized protocol to test rabbit sera immunized with PBS (negative control), low dose of Influenza A vaccine with adjuvant, high dose vaccine, and high dose vaccine with adjuvant. When comparing normalized peptide counts from serum samples with those from beads-only controls, enriched Influenza A virus peptides (represented as black diamonds) are seen present in each sample (vaccinated and unvaccinated), but a clear visual increase in the number and normalized count in the vaccinated samples (Figure 3A).

We first sought to quantify this difference globally but found no significant difference (Figure 3B). However, differences between vaccinated and unvaccinated samples became clearer when the enriched Influenza A virus peptides were further analyzed. The protein-level breakdown of enriched Influenza A virus peptides for all samples included hemagglutinin, NSP, and neuraminidase (Figure 3C(i)), but when the normalized count of each peptide is accounted for, enrichment predominantly corresponds to hemagglutinin peptides, with even greater enrichment seen with adjuvanted samples (Figure 3C(ii)).

This hemagglutinin target was then analyzed closer for epitope-level specificity (Figure 3D). The peptide-level breakdown of enriched hemagglutinin peptides based on total normalized count revealed one dominant peptide among vaccinated samples, corresponding to the vaccine-specific serotype and confirmed cross-reactive strain: H5N1 Hong Kong/2002. Beyond the dominant peptide, the top 5 peptides all correspond to the same region with two overlapping peptide targets, each showing strong similarity in sequence to the vaccine and cross-reactive strains (Figure 3G).

In order to get a clearer picture of the overall antibody HA-specific repertoire of these samples beyond the top hits (similar to Figure 2), we characterized the HA protein reactivity amongst all vaccinated samples as a colormap. The relative hemagglutinin enrichment was plotted on a residue-level heatmap normalized to the non-vaccinated control (Figure 3E) and revealed distinct epitopes across vaccinated samples with common epitopes between all three samples (residues 421–531) and the low dose plus adjuvant and unadjuvanted high dose samples (residues 81–111, 281–341). Notably, a 3D visualization of relative enrichment (Figure 3F) illustrates the dominant epitope on the stem of the protein (29), a plausible target overlapping with a common public linear epitope identified in the Immune Epitope Database (30).

**FIGURE 4:**
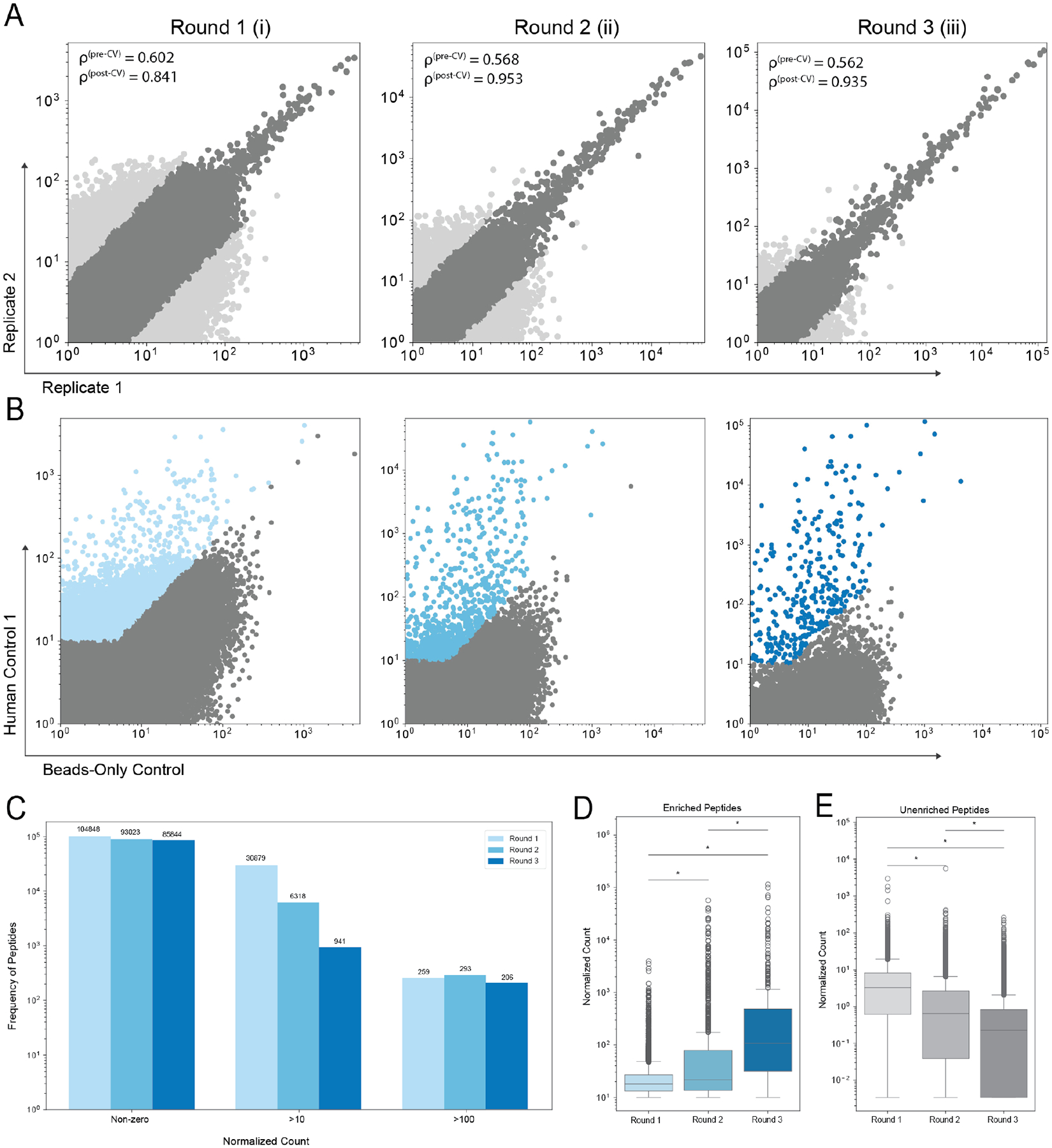
Three rounds of immunoprecipitation increase signal of enriched peptides. (**A**) Peptide outliers are excluded in CV thresholding of serum replicates. (**B**) Normalized counts of remaining peptides from serum sample and beads-only control after each round of immunoprecipitation (enriched peptides with count > 10 and foldchange > 2 in color, non-enriched in grey). (**C**) Representation of phage library at each round of immunoprecipitation, grouped by normalized count (non-zero, over 10, and over 100). (**D**) Distributions of normalized counts of enriched peptides after each round of immunoprecipitation. (**E**) Distributions of normalized counts of non-enriched peptides after each round of immunoprecipitation. * Means: P value < 0.05.

Finally, in order to identify balance between enrichment and library representation using our optimized version of iterative PhIP-seq, we analyzed the results of three successive rounds of capture. We found similar replicability between samples (as measured by Spearman’s rank correlation coefficient (ρ), both pre- and post-CV thresholding, respectively: 0.602, 0.568, and 0.562; and 0.841, 0.953, and 0.935) (Figure 4A) and an increase in enrichment of peptides, most prominently increased upon three captures. This was quantified by significant improvement in the normalized counts of enriched peptides after three captures (Figure 4D). There was also a notable decrease in the normalized counts of the non-enriched peptides after each round (Figure 4E). This suggests that three subsequent captures provide significantly more signal and less noise by a metric of normalized count over a 2-round and single-round approach.

Surprisingly, analysis of library representation upon three captures revealed that no significant reduction of library representation occurs (Figure 4C). Fittingly, the distribution of phage enrichment broadens considerably upon three captures, but the total representation allows the possibility that three captures still enable global depiction of an antibody repertoire. Notably, a decrease is seen in the number peptides with normalized counts between 10 and 100 after each additional round of IP, reflecting both a potential for a loss in signal from more moderate/weak peptide hits and—critically to this work—an increased separation between enriched peptides and background noise.

## Discussion

Overall, we present a method of optimizing the VirScan application of the PhIP-seq protocol to maximize signal-to-noise ratio through additional rounds of immunoprecipitation and pre-washing sera with wildtype phage. Both modifications significantly increased the signal of enriched peptides as measured by an increase in their normalized count. Together, the effect was compounded, supporting the conclusion that the two modifications worked synergistically to increase the signal-to-noise ratio via the iterative honing of the input library to more closely represent a pool of sample-specific antibody-targeted peptides while minimizing non-specific binding. The term “noise” refers to the phages that were indiscriminately pulled down or remained after the wash steps, leading to their eventual sequencing and inclusion in the dataset.

These refinements to the VirScan workflow yielded raw counts with improved identification of an enriched population in all serum samples. Only with clinical validation can the correlation between increased enrichment of peptide hits and accurate detection of viral history be linked. To approach this in a curated fashion, we validated seropositivity with animal influenza specimens and human HIV specimens from a publicly available source. With the pooled human HIV seropositive sera, the ability of our optimized protocol was confirmed to be able to detect broad seropositivity when compared to human control serum, and the dependence on antibody concentration was shown. Multiple immunodominant regions were identified for both of these conditions. With the vaccinated samples, the ability of our optimized protocol to detect strain-specific vaccine-elicited antibody specificities was confirmed, additionally being able to detect differences between adjuvanted and non-adjuvanted vaccinated samples via the strength of the target epitope “hit.”

In determining the optimal number of rounds of immunoprecipitation to perform to increase signal of true peptide hits, the risk of bottlenecking was considered. While in theory, each additional round would further increase the likelihood that the phage pool accurately represents true antibody targets, it also introduces a new bottlenecking event as the whole amplified library is not used in the next round of immunoprecipitation. This presents the risk of losing sensitivity in the assay. While three rounds were shown to maintain library diversity, indicating that broad repertoire sensitivity would still be possible, the effect of further additional rounds were not tested. It is possible that it may lead to further separation of signal from noise, but the potential effect on sensitivity should not be ignored. The labor required for three rounds of precipitation is significantly more than that required for one or even two rounds. Depending on scale and scope of prospective research, three rounds may not be ideal or feasible due to cost, labor, or the potential for lost sensitivity. The advantages and disadvantages to the original and optimized approach are outlined in Table 1.

This work provides routes of improvement for common obstacles faced in PhIP-seq research, specifically that of excessive noise, but does not overcome other limitations inherent with the method. Primarily, PhIP-seq cannot replicate ELISA-level results on a high-throughput scale, with the additional variables of unequal library representation, inter-phage binding competition, and other nonspecific binding opportunities with beads creating more variability than normally accounted for in the ELISA due to differential affinity and avidity of all antibody-antigen pairs. These variables make any internal controls (such as an externally confirmed mAb spike in) problematic. Thus, our definition of an “enriched peptide” is made up from arbitrary thresholds developed to create a measure from which the effect of the various modifications tested in this work could be quantified easily. The translation from epitope “hit” to seropositivity must be tread with caution and often relies on external confirmation or at the very least, public epitope cross-referencing. As shown in Figures 2A, the enrichment thresholds used in this work are imperfect, as HIV peptides exceed this cutoff in our healthy human control. Our thresholding was benchmarked against the previously published PhIP-Stat pipeline (3), showing a substantial overlap in what the two consider “enriched”, while each pipeline does consider some peptides enriched that the other does not, our pipeline appears not to be limited greatly by our use of a CV threshold between replicates (Supplementary Figure 4). Detection of unexpected viral peptides in control samples necessitates additional contextual analysis to distinguish true signal from noise, In the case of HIV, comparison of the control sample with HIV-positive serum dilutions (Figure 2C, D) reveals a clear separation in signal magnitude. Additionally, HIV ranks fifth in total peptide representation within the VirScan library (Supplementary Table 2), increasing the probability of stochastic and low level signal detection. A similar pattern is observed for Influenza A peptides (Figure 3A); however, further analysis (Figures 3B–E) demonstrates a clear difference in enrichment of Influenza A peptides between vaccinated and unvaccinated samples.

Expanding on the issue of thresholding, the construction of a PhIP-seq analysis pipeline requires numerous decisions related to filtering and data interpretation. The complexity of the VirScan library, with the inclusion of multiple strains of virus, isoforms of proteins, and polyproteins, and potential cross-reactive epitopes on unrelated viral peptides makes the analysis of a peptide region exceptionally difficult. A target protein could be represented many times across the library in different forms, such as the standalone protein or as part of a larger polyprotein (like gp160 used for HIV analysis). Various analytical pipelines exist to address this issue, each with their own assumptions and/or thresholding choices, including z-score thresholding and viral score construction based on rank-ordered viral assignment (11) and AVARDA—an algorithm that carefully considers antibody cross-reactivities across similar viruses while accounting for disproportionate library representation (20).

Nevertheless, these analytical pipelines cannot overcome certain inherent challenges of PhIP-seq. Displayed peptides are one-dimensional, lack post-translational modifications, and library amplification contains potential for representation bias. Beyond library design, validation strategies remain an obstacle. Thus far VirScan validation has been made on clinical data (4) with the help of validation ELISAs (7, 31). To validate new and high throughput hits from VirScan, a large amount of pre-validation must be done. This “pre-work” has not yet been implemented in our protocol, but others have been working towards it (5). Establishment of a solid positive control is useful for validating VirScan data, however the issue with using a positive control as a marker of a definite hit is the variance between peptides in the library. Countless factors affect the binding affinity and behavior of each peptide to its corresponding antibody. Some peptides within the library seem to bind indiscriminately to antibody these peptides are noted, but without a completely characterized human control sample, it is additional work to validate that these peptides are indeed a false hit.

This work highlights practical strategies for reducing avoidable noise in VirScan data and provides simple analytical steps for assessing data quality and reproducibility. The considerations brought up in this work underscore that PhIP-seq is most powerful when used with a clear understanding of its limitations and is interpreted using context-aware frameworks.

## Methods

### Amplification and titering of VirScan phage library

Plaque assays and plate amplification protocols were adapted from the Novagen T7Select System Manual (32). Approximately 2.5 × 10^7^ pfu of phage library was added to 50 mL BLT5403 E. coli, at OD600 10. This was plated on 16 12” × 12” LB Carb plates, mixing with 45 mL top agar for each plate. Plates were incubated at 37 °C until complete clarification (after approximately 4 hours), at which point 30 mL Phage Extraction Buffer were added per plate. The plates were then rocked overnight at 4C before harvesting phage and titering with a plaque assay.

### PhIP-seq protocols

PhIP-seq comparison experiments (Figure 1C(i–iv)) were conducted according to previously published protocols (Original, Phage-Antibody Optimized, and Multi-IP) with the following modifications: 5 μL of Pierce Protein A/G magnetic beads were used per sample after blocking overnight in 5% BSA in TNP40, complexing between antibodies and beads was performed prior to adding the phage library, and all PCRs were performed using 2X Q5 Master Mix (11, 33). Pre-washed samples were tested using the same modified multiple IP protocol as used in the comprehensive method comparison with the addition of a wildtype phage serum pre-wash. In the first round of IP, the antibody–bead complexes were incubated with 500 μL 4.6 × 10^9^ pfu/mL wildtype T7 phage for one hour before the VirScan library was added. The optimized PhIP-seq protocol was based on the modified multiple IP protocol with pre-wash as described above with the following changes: 40 μL of blocked Pierce Protein A/G magnetic beads were used for each IP and sera was complexed to phage overnight before the addition of the beads. All samples were run in duplicate. All experiments were run in parallel with a mock-IP control, which simulates antibody independent phage to bead binding.

### Data analysis

The bulk processing of sequencing results was conducted in accordance with a previously published pipeline (11), resulting in count files displaying the peptide counts for each member of the VirScan peptide library for each sample. These counts were then normalized to sequencing depth (count/million reads), and low-replicability peptides (peptides whose coefficient of variability value, CV, was >= 100) were removed from each sample file before replicate counts were averaged and used to identify enriched peptides. An enriched peptide was defined as any peptide with sufficient replicability (CV < 100), a fold change over bead control of at least 2, and a normalized count of at least 10.

## Supplementary Material

Supplementary Material **SUPPLEMENTARY FIGURE 1** Phage library diversity maintained post-amplification. (left) Frequency of non-zero peptide counts after sequencing phage library post-amplification (normalized to sequencing depth). (right) Total peptide representation pre- and post- amplification.
**SUPPLEMENTARY FIGURE 2** PCA analysis of sample replicates and mock-IP samples. The principal component analyses of peptide hits before and after CV filtering of bead only control and patient 1 sample (each replicate shown individually) for Original protocol (**A**), Phage-AB optimized (**B**), Multi-IP (**C**), Multi-IP + Pre-wash (**D**), and the optimized protocol (**E**). Principal component 1 is a weighted average of all peptides combined in a way to show the maximum possible variance in the data. Principal Component 2 captures subtler differences usually representing things such as differences between replicates.
**SUPPLEMENTARY FIGURE 3** Top Virus Hits for Human Control Serum 1. (**A**) The top 10 most abundant viruses by normalized count for each method. (**B**) The top 10 viruses by the number of enriched peptides for each method.
**SUPPLEMENTARY FIGURE 4** A comparison of enrichment profiles on Human Control 1 using both our analysis pipeline and the previously established PhIP-Stat pipeline. Replicate variation is shown in normalized counts with an overlay highlighting the peptides that meet the enrichment criteria for our pipeline, the PhIP-Stat pipeline or both (**A**), the same overlay is applied when Human Control 1 normalized count is plotted against that of the bead only control. (**B**). The enrichment profiles of each pipeline are shown, highlighting the overlap of the enrichment criteria. (**C**) Of the 116 peptides that PhIP-Stat includes but our code does not, 5 were excluded due to our CV < 100 threshold, and the other 111 peptides did not meet our log-fold change > 2 requirement and were therefore excluded.
**SUPPLEMENTARY TABLE 1** Peptide exclusion by CV filter. Sorted by protocol optimization: total library peptides detected, peptides excluded by CV filtering, retained within the CV threshold, and the percentage of peptides excluded by our CV threshold.
**SUPPLEMENTARY TABLE 2** Compiled list of the 50 viruses with the largest peptide representation within the VirScan library.

The Supplementary Material for this article can be found online at: https://www.frontiersin.org/articles/10.3389/fviro.2026.1761741/full#supplementary-material

## Figures and Tables

**TABLE 1:** Comparison of strengths and limitations of both the original PhIP-seq protocol and our optimized PhIP-seq protocol.

	Original PhlP-seq protocol	Optimized protocol
Strengths	Scalability: The single IP workflow is faster, cheaper, and less labor-intensive. Retention of low affinity interactions: A single round of IP allows for a more diverse picture of antibody repertoire, allowing for the detection of weak but possibly biologically relevant antibodies. Established standard: It is a well characterized workflow used in existing literature.	Specificity: Pre-wash and Multi IP drastically reduce non-specific binding. Reproducibility: The requirement for a peptide to enrich across multiple rounds filters out non-specific binding events. The retained hits are robust, high-affinity interactions that can be easily separated for further analysis.
Limitations	Noise: Non-specific binding to beads or phage creates a large amount of background, making it difficult to detect true hits. False Positives: Sticky peptides can repeatedly appear as hits due to consistent non-specific binding. Signal ambiguity: It can be hard to distinguish a true, weak binding event from noise.	Bottleneck: The conditions of the optimized protocol may lead to the loss of signal from biologically significant, low affinity peptide-AB interaction. Resource use: The multi-IP approach and high bead usage increase labor and reagent costs significantly. Complexity: The extensive handling of samples introduces increased risk for technical error if not managed properly.

## Data Availability

The original contributions presented in the study are publicly available. This data can be found here: NCBI Sequence Read Archive, BioProject accession PRJNA1438256.
