# Supplementary material for: Iterative immunoprecipitation and phage pre-wash dramatically improve epitope-resolved serology by VirScan
Source: Front Virol. Author manuscript; Available in PMC 2026 May 15. (PMC13175123; doi:10.3389/fviro.2026.1761741)
Supplement: Supplementary Material — SUPPLEMENTARY FIGURE 1 Phage library diversity maintained post-amplification. (left) Frequency of non-zero peptide counts after sequencing phage library post-amplification (normalized to sequencing depth). (right) Total peptide representation pre- and post- amplification. SUPPLEMENTARY FIGURE 2 PCA analysis of sample replicates and mock-IP samples. The principal component analyses of peptide hits before and after CV filtering of bead only control and patient 1 sample (each replicate shown individually) for Original protocol (A), Phage-AB optimized (B), Multi-IP (C), Multi-IP + Pre-wash (D), and the optimized protocol (E). Principal component 1 is a weighted average of all peptides combined in a way to show the maximum possible variance in the data. Principal Component 2 captures subtler differences usually representing things such as differences between replicates. SUPPLEMENTARY FIGURE 3 Top Virus Hits for Human Control Serum 1. (A) The top 10 most abundant viruses by normalized count for each method. (B) The top 10 viruses by the number of enriched peptides for each method. SUPPLEMENTARY FIGURE 4 A comparison of enrichment profiles on Human Control 1 using both our analysis pipeline and the previously established PhIP-Stat pipeline. Replicate variation is shown in normalized counts with an overlay highlighting the peptides that meet the enrichment criteria for our pipeline, the PhIP-Stat pipeline or both (A), the same overlay is applied when Human Control 1 normalized count is plotted against that of the bead only control. (B). The enrichment profiles of each pipeline are shown, highlighting the overlap of the enrichment criteria. (C) Of the 116 peptides that PhIP-Stat includes but our code does not, 5 were excluded due to our CV < 100 threshold, and the other 111 peptides did not meet our log-fold change > 2 requirement and were therefore excluded. SUPPLEMENTARY TABLE 1 Peptide exclusion by CV filter. Sorted by protocol optimization: total library peptides detecte [file NIHMS2167889-supplement-Supplementary_Material.docx]

Supplemental Figure 1: Phage library diversity maintained post-amplification. (left) Frequency of non-zero peptide counts after sequencing phage library post-amplification (normalized to sequencing depth). (right) Total peptide representation pre- and post- amplification.


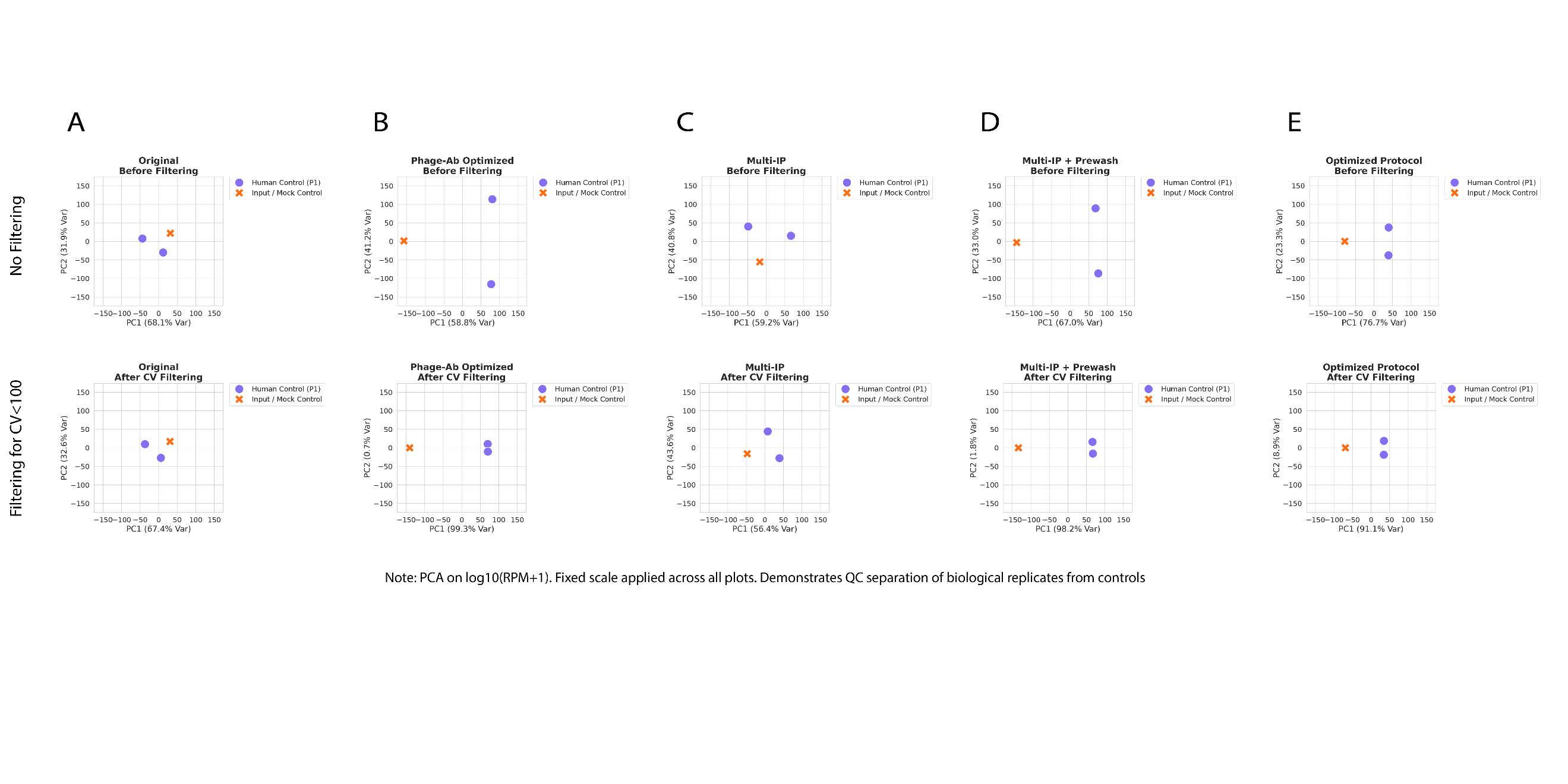


Supplementary Figure 2: PCA analysis of sample replicates and mock-IP samples. The principal component analyses of peptide hits before and after CV filtering of bead only control and patient 1 sample (each replicate shown individually) for Original protocol (A), Phage-AB optimized (B), Multi-IP (C), Multi-IP + prewash (D), and the optimized protocol (E). Principal component 1 is a weighted average of all peptides combined in a way to show the maximum possible variance in the data. Principal Component 2 captures subtler differences usually representing things such as differences between replicates.


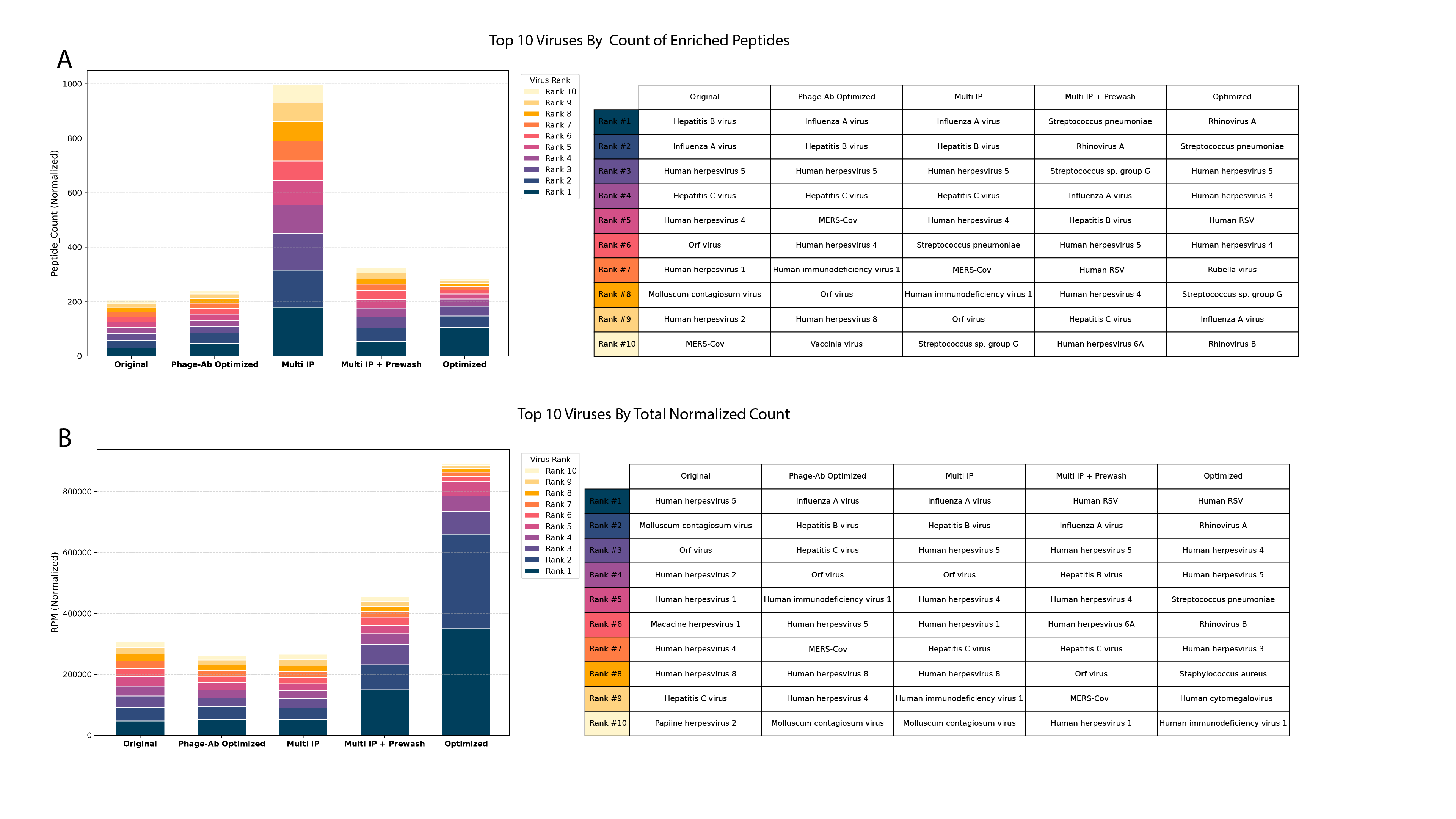


Supplementary Figure 3: Top Virus Hits for Human Control Serum 1. A) The top 10 most abundant viruses by normalized count for each method. B) The top 10 viruses by the number of enriched peptides for each method.


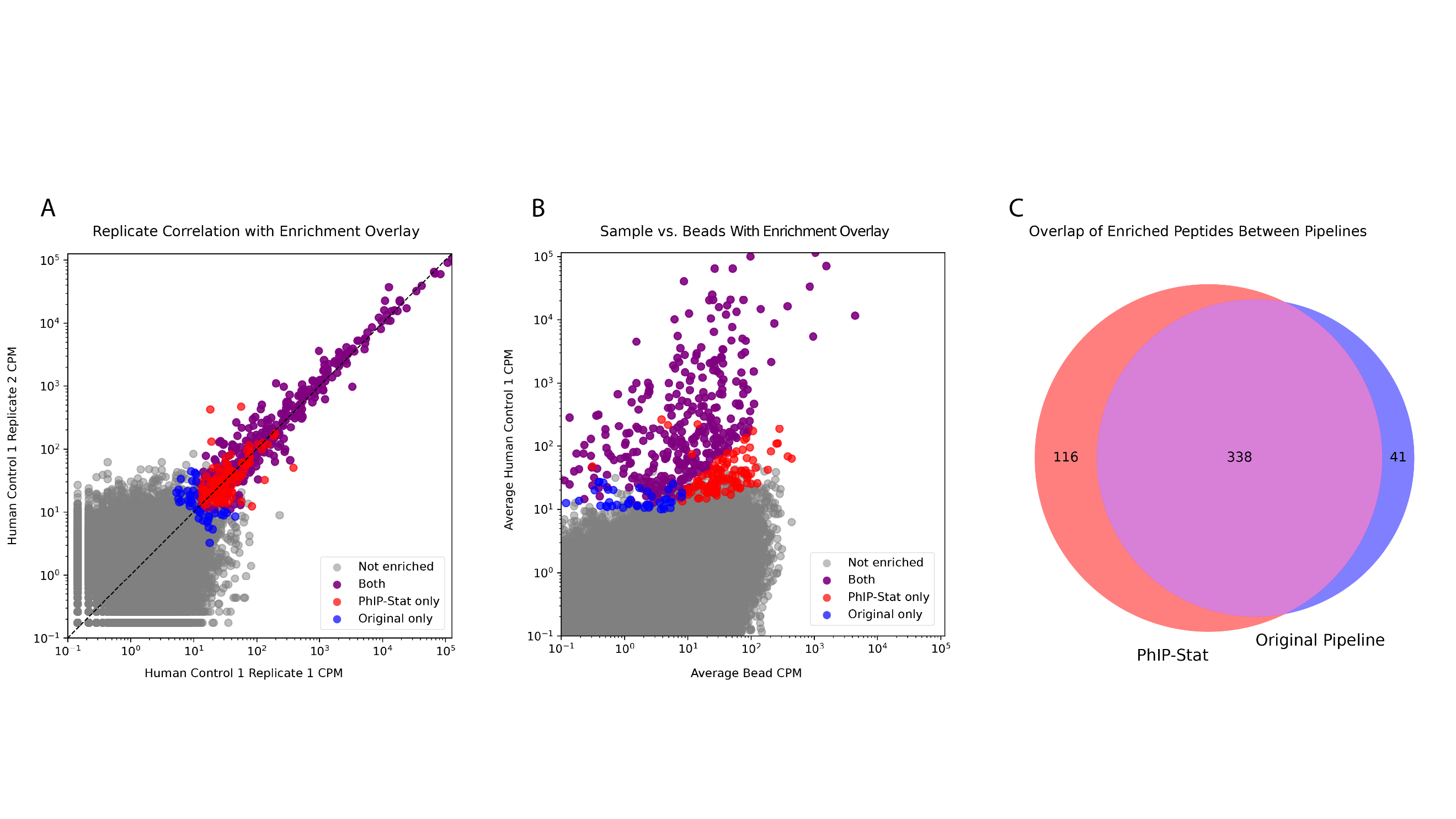


Supplementary Figure 4: A comparison of enrichment profiles on Human Control 1 using both our analysis pipeline and the previously established PhIP-Stat pipeline. Replicate variation is shown in normalized counts with an overlay highlighting the peptides that meet the enrichment criteria for our pipeline, the PhIP-Stat pipeline or both (A), the same overlay is applied when Human Control 1 normalized count is plotted against that of the bead only control. (B). The enrichment profiles of each pipeline are shown, highlighting the overlap of the enrichment criteria. (C) Of the 116 peptides that PhIP-Stat includes but our code does not, 5 were excluded due to our CV < 100 threshold, and the other 111 peptides did not meet our log-fold change > 2 requirement and were therefore excluded.


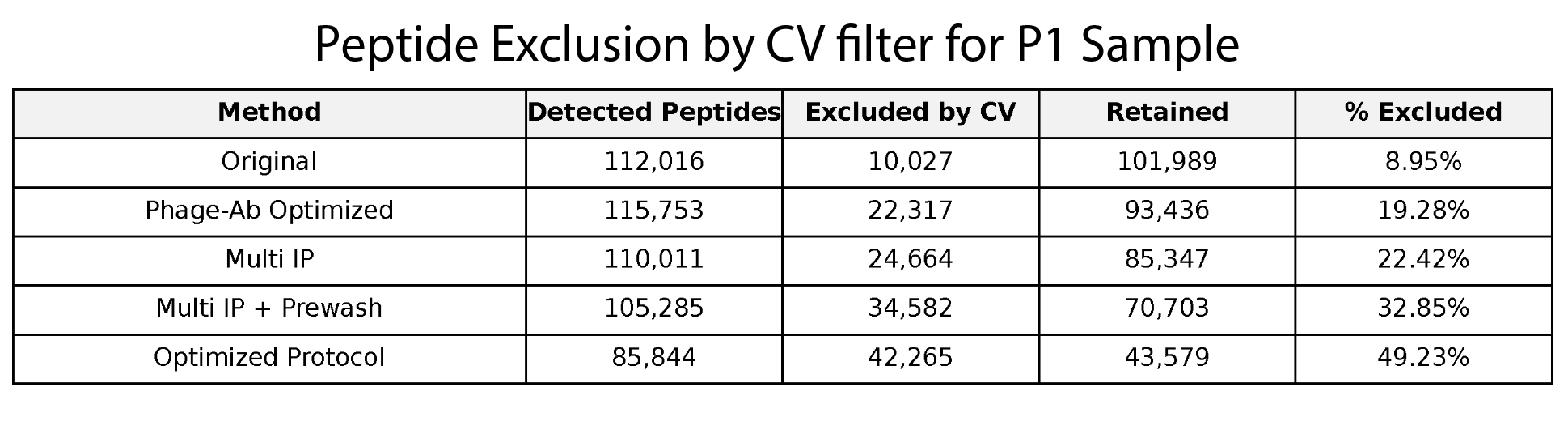
Supplementary Table 1: Peptide Exclusion by CV Filter. Sorted by protocol optimization: total library peptides detected, peptides excluded by CV filtering, retained within the CV threshold, and the percentage of peptides excluded by our CV threshold.


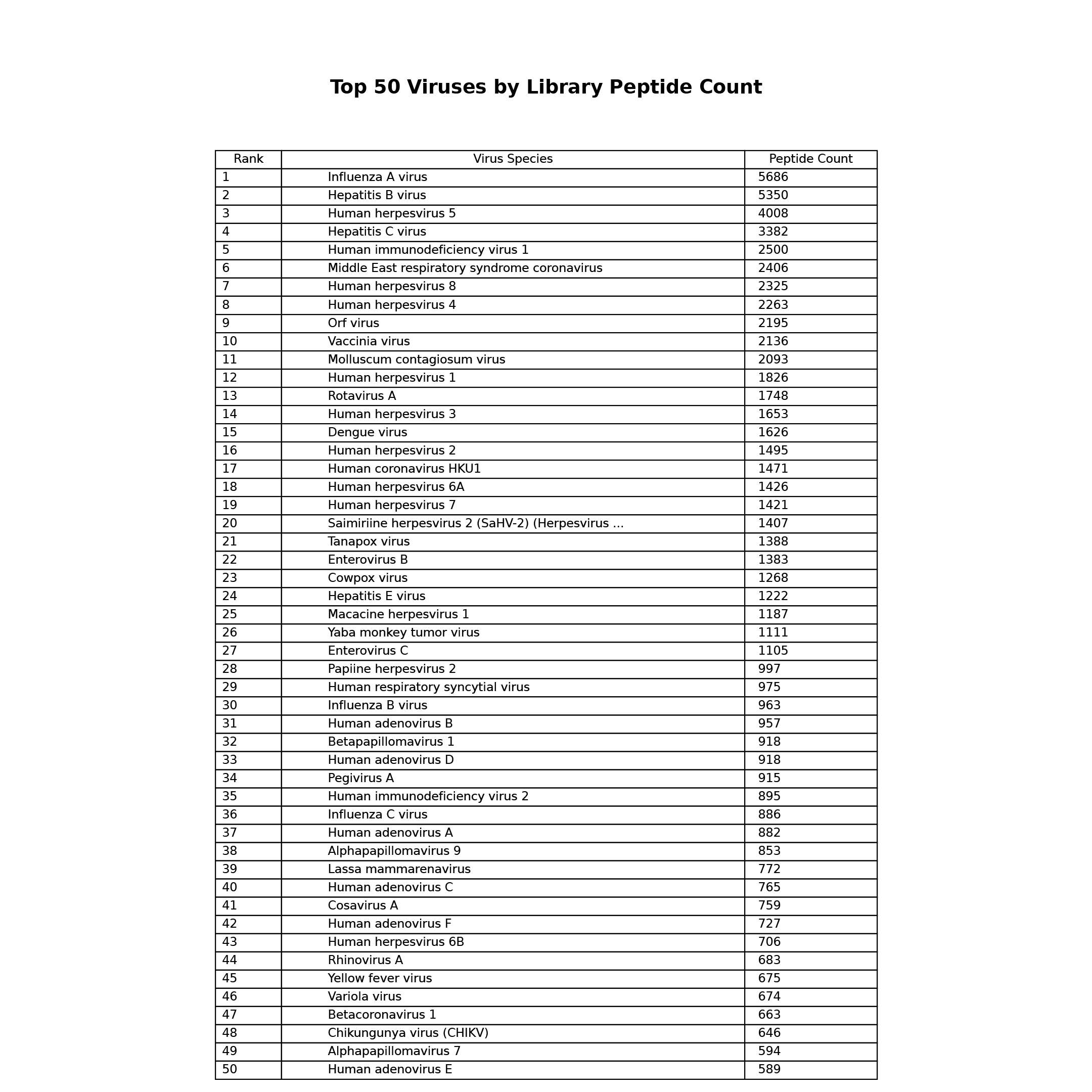


Supplementary Table 2: Compiled list of the 50 viruses with the largest peptide representation within the VirScan library.
